# Dual-Task Abilities During Activities Representative of Daily Life in Community-Dwelling Stroke Survivors: A Pilot Study

**DOI:** 10.3389/fneur.2022.855226

**Published:** 2022-05-03

**Authors:** Anne Deblock-Bellamy, Anouk Lamontagne, Bradford J. McFadyen, Marie-Christine Ouellet, Andréanne K. Blanchette

**Affiliations:** ^1^Faculty of Medicine, Universite Laval, Quebec City, QC, Canada; ^2^Center for Interdisciplinary Research in Rehabilitation and Social Integration (Cirris)–CIUSSS de la Capitale-Nationale, Quebec City, QC, Canada; ^3^School of Physical and Occupational Therapy, McGill University, Montreal, QC, Canada; ^4^Jewish Rehabilitation Hospital-CISSS de Laval, Centre for Interdisciplinary Research in Rehabilitation of Greater Montreal (CRIR), Laval, QC, Canada; ^5^Department of Rehabilitation, Universite Laval, Quebec City, QC, Canada; ^6^Faculty of Social Sciences, School of Psychology, Universite Laval, Quebec City, QC, Canada

**Keywords:** stroke, dual-task, interference, locomotion, cognition

## Abstract

**Background:**

In addition to several physical skills, being able to walk in the community, walking independently and safely in the community requires the ability to divide attention between walking and other tasks performed simultaneously. The aims of the present pilot study were to measure cognitive-locomotor dual-task (DT) abilities during activities representative of daily living in stroke survivors and to compare them with age- and gender-matched healthy individuals.

**Methods:**

To assess DT abilities, all participants walked along a virtual shopping mall corridor and memorized a 5-item shopping list. Two levels of task complexity were used for the walking task (with or without virtual agents to avoid) and the cognitive task to recall a list of items (with or without a modification at mid-course). The assessment was conducted using an omnidirectional platform and a virtual reality (VR) headset. Locomotor and cognitive DT costs (DTC) were calculated as the percent change from single-task (ST) performance. Walking speed and minimal distance between the participant and the virtual agents were used to characterize locomotor performance. Cognitive performance was assessed by the number of correctly recalled items. One-sample Wilcoxon tests were used to determine the presence of DTCs and Mann-Whitney tests were performed to compare DTCs between the 2 groups.

**Results:**

Twelve community-dwelling stroke survivors [60.50 years old (25-75^th^ percentiles: 53.50–65.75); 5 women; 13.41 months post-stroke (5.34–48.90)] and 12 age- and gender- matched healthy individuals were recruited. Significant cognitive or mutual (cognitive and locomotor) interferences were observed in participants with stroke in all DT conditions, except the simplest (no virtual agents, no modifications to the list). For the control group, significant mutual interferences were only observed during the most complex DT condition. A group difference was detected in cognitive DTCs during the most complex DT condition (virtual agents and list modifications; *p* = 0.02). Stroke survivors had greater cognitive DTCs than the control group.

**Conclusions:**

Using an ecological perspective contributes to understanding behavior of stroke survivors in daily activities. Virtual scenarios appear to be an interesting avenue for a more comprehensive understanding of DT abilities during activities representative of daily living in stroke survivors. The usability and feasibility of such an approach will have to be studied before considering implementation in rehabilitation settings.

## Introduction

The ability to get out and about in the community is considered very important, even essential, for 73% of stroke survivors (1). Walking in the community provides a sense of freedom and control over one's own life (2). However, one study indicated that, on discharge from an inpatient rehabilitation setting, only one-quarter of stroke survivors had the physical capacity to walk independently in the community (3). As a result, community walking may be associated with negative feelings about walking difficulties (2).

Walking independently and safely in the community is a complex activity that requires several capacities including minimal walking speed and endurance, as well as the ability to negotiate stairs or to walk on uneven ground (4, 5). In addition, community walking also demands the ability to divide attention between walking and other tasks performed simultaneously (6) such as talking with a friend, paying attention to store signs, or reading a text message on a smartphone.

Performing a cognitive task while walking (dual tasking) often results in performance deterioration in one or both tasks, when compared to the performance of each task assessed separately (single task; ST). Cognitive-motor interference during walking is believed to reflect competing demands on limited cognitive processes (7, 8). Since dual task (DT) can induce changes in both locomotor and cognitive performances, different cognitive-motor interference patterns can be observed. Plummer et al. (9) have defined several patterns of DT interference among which the most frequently observed are: deterioration of motor and cognitive performances (mutual interference), deterioration of motor performance only (motor interference), deterioration of cognitive performance only (cognitive interference), and no change in performance for both tasks related to ST performance change (no interference). These DT patterns depend on several factors, such as the nature and the difficulty of both tasks (10–12), the environment in which the tasks are performed (13, 14) as well as on participant characteristics such as age (15, 16) or the presence of a neurological pathology (9, 10, 17).

Several studies have examined the impact of a stroke on DT abilities using different combinations of cognitive and locomotor tasks [reviewed in (18)]. This literature review has shown that cognitive-motor interference has been mainly explored using protocols including simple locomotor tasks and cognitive tasks that are not representative of activities of daily living such as an arithmetic task (e.g., subtractions) or a Stroop task. Thus, it is currently difficult to estimate the impact of performing two tasks simultaneously on daily activities in stroke survivors. To improve the ecological validity of assessments, recent studies have explored DT abilities of stroke survivors in a real-life environment (19, 20). Assessing DT abilities with a standardized and reproducible protocol in the community remains a challenge. To overcome this issue, virtual reality (VR) holds promise as it can provide standardization of protocols, and measures can be taken to prevent falls. A few teams have in fact successfully used VR to assess and train DT locomotor ability in stroke survivors (21–23). In the present pilot study, a VR-based protocol was developed to assess DT abilities during activities representative of daily life in a standard and safe manner. The objectives of the present pilot study were (1) to measure locomotor and cognitive DT abilities in stroke survivors in conditions with different levels of complexity, and (2) to compare them with those measured in age-matched healthy individuals. We hypothesized that stroke survivors would exhibit locomotor and cognitive DT cost (DTC) during activities representative of daily life and that these would be greater than those measured in age-matched healthy individuals.

## Methods

### Participants

Community-dwelling stroke survivors and healthy participants, matched for age and gender, were recruited on a voluntary basis. Stroke survivors with moderate to severe cognitive impairment [Montreal Cognitive Assessment score <22, MoCA (24)], visuospatial neglect as assessed by both the Star Cancellation Test [cutoff score: <52/54 (25)] and the Apple Test [cutoff score: <42/50 (26)], moderate to severe aphasia affecting the understanding of the task to be performed, any locomotor disorder not related to the stroke, or any other neurological disorder were excluded. Age- and gender-matched healthy participants were excluded if they had cognitive impairments [MoCA < 26], any neurological disorder or locomotor deficit that could affect walking during assessment. Before participating in the study, all individuals read an information sheet and signed a consent form. This study was approved by the Institutional Ethics Committee of the Centre Intégré Universitaire de Santé et de Services Sociaux de la Capitale-Nationale [CIUSSS de la Capitale-Nationale; # 2019-1720].

### General Procedure

**Figure 1:**
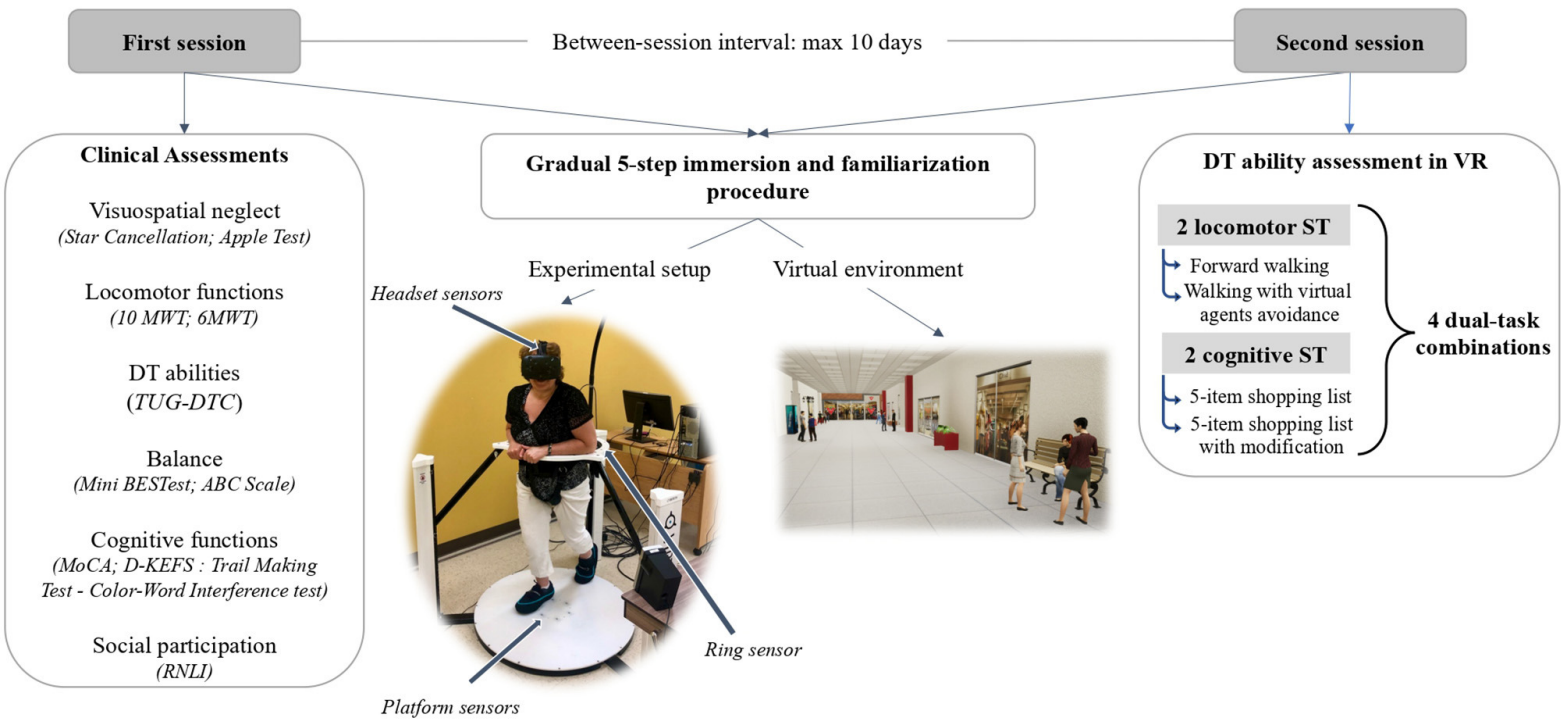
Experimental setup. 6MWT, 6-min Walk Test; 10MWT, 10-meter Walk Test; ABC Scale, Activities-Specific Balance Confidence Scale; D-KEFS, Delis-Kaplan Executive Functions System; DT, dual-task; Mini BESTest, Mini Balance Evaluation Systems Test; MoCA, Montreal Cognitive Assessment; ST, single task; RNLI, Reintegration of Normal Living Index; TUG-DTC, Dual-task cost of Timed-Up Go; VR, virtual reality.

All participants took part in two assessment sessions, each lasting approximately 90 min, to the Centre for Interdisciplinary Research in Rehabilitation and Social Integration (Cirris) of the CIUSSS de la Capitale-Nationale. The first session was dedicated to ensuring participant's eligibility (visuospatial neglect tests for all stroke survivors and MoCA for all participants), collecting sociodemographic (age, gender, occupation and leisure activities) and general health data using home-made questionnaires. In addition, walking, balance and cognitive functions as well as DT abilities and social participation were assessed with clinical assessment tools. These assessments were conducted by experienced neurological rehabilitation professionals. The first session ended with a gradual 5-step immersion and familiarization procedure (described below), so that the participants could get used to the equipment and the virtual environment that would be used in the second session. The second assessment session was mainly dedicated to measure cognitive-locomotor DT abilities using VR-based protocol. However, the immersion and familiarization procedure was repeated at the very beginning of this visit (Figure 1).

### Clinical Outcome Measures

Several clinical assessments were used to describe participant's abilities. Gait function was quantified using the 10-meter Walk Test [10MWT (27)] and the 6-min Walk Test [6MWT (28)] to measure preferred/maximal speeds and endurance, respectively. To assess balance, participants performed the Mini Balance Evaluation Systems Test [Mini BESTest (29)]. In addition, participants completed a French-Canadian version of the Activities-Specific Balance Confidence Scale [ABC Scale (30)] to assess their confidence in performing various activities without losing balance.

Cognitive inhibition and flexibility skills were evaluated using two subtests of the Delis-Kaplan Executive Functions System (D-KEFS) i.e., the Color-Word Interference and the Trail Making Test (31, 32). The raw scores in seconds were then converted in scaled-score equivalents by each age group (max.: 19).

To mitigate the impact of variability in naming and reading skills during the Color-Word Interference Test, the contrast score was used in the present study. It was calculated by the subtraction of the combined scaled score of “color naming + word reading” conditions from “inhibition-switching” condition (switching between naming the color ink and reading the color word). For the Trail Making Test, the contrast score subtracting the scaled score of the “motor speed” condition from the scaled score of the “number-letter switching” condition was used to reduce the impact of variability in motor skills. For these two D-KEFS subtests, the scale scores have a mean of 10 and a standard deviation of 3 (31). A score below 7 indicates the presence of a cognitive impairment.

To quantify the impact of poststroke limitations on social participation, stroke survivors completed a French version of the Reintegration of Normal Living Index [RNLI (33)]. The tool includes 11 items scored on a 3-point scale covering participation in recreational and social activities. Total scores range from 22 to 0. The higher the score, the worse the reintegration of stroke survivors (34).

### Cognitive-Locomotor DT Assessment

DT abilities were first measured using a standard clinical assessment, i.e., the Timed Up and Go (TUG) in single (TUG-ST) and dual tasks (TUG-DT). In the TUG-ST, participants were required to stand up from a chair, walk 3 meters, turn around, and return to sit. For the TUG-DT, participants performed the same locomotor task while counting backwards by 3 from a randomly-selected number between 20 and 100. The time taken to perform TUG-DT was subtracted from TUG-ST. The locomotor DT cost (TUG-DTC) was then calculated as a % of mean ST duration (35, 36). A negative TUG-DTC indicates that task duration increased in DT condition.

DT abilities were also quantified using an experimental standardized protocol which assesses locomotor and cognitive DTCs during activities representative of everyday life in a virtual environment. This VR-based DT assessment protocol has previously been used to quantify DT abilities in healthy young adults (37).

DT abilities were assessed in a 75-m virtual straight corridor of a shopping center. The virtual environment was created in Unreal Editor 4.19.2 (Epic Game, Cary, North Caroline, USA). The experimental setup consisted of a low-friction omnidirectional platform with a rotating ring attached tightly to a harness worn at the pelvis level (Virtualizer, Cyberith GmbH, Vienna, Austria) and a VR headset (Vive, HTC Corporation). Progression in the virtual environment was enabled by 6 sensors located in the center of the platform. A sensor in the ring combined with the headset sensors were used to track the participant's orientation. To walk on the platform, participants were secured in a harness fixed on the ring. No body weight support was provided with the harness, but participants had the option to put their upper limbs on the ring. For participant safety, the omnidirectional platform included a mechanical stop system preventing falls (Figure 1).

DT abilities were assessed in 4 combinations of DT, composed of two locomotor and two cognitive tasks of different levels of complexity.

#### Locomotor Tasks

- Simple: 75-m forward walking without virtual agent avoidance.
- Complex: 75-m forward walking with 3 virtual agents to avoid. Regardless of their initial position within the environment, each virtual agent was programmed to walk into the path of the participant and begin walking toward them at a point corresponding to 7 m in front of the participant. The 3 virtual agents arrived at different times along the 75 m path and walked at the participant's speed or at a minimal speed of 1.2 m/s.

#### Cognitive Tasks

- Simple: Memory task of a 5-item shopping list orally delivered at the beginning of the trial. At the end of the trial, participants had to verbally recall the shopping items.
- Complex: Memory task of a 5-item shopping list orally delivered at the beginning of the task with a verbal modification of 2 items in the middle of the walking course. At the end of the task, participants had to verbally recall the modified list of items.

A different list of items was used on each trial and each list targets a specific type of store (e.g., grocery, pharmacy, hardware store, clothing store). In the ST condition, both cognitive tasks were performed while sitting in both the real and virtual environments.

To limit the occurrence of cybersickness and familiarize participants with the locomotor task on the omnidirectional platform, a gradual 5-step immersion and familiarization procedure was carried out with all participants: (1) use eye movements to explore the environment without moving the head or body; (2) use head movements to explore the environment without moving the rest of the body; (3) perform trunk movements to explore the environment, without walking in the virtual environment; (4) move forward in the virtual environment and (5) walk approximately 100 m in a virtual shopping mall environment, including 3 changes of direction and 2 virtual agent avoidances. In case of collision, in the last step, participants had to retry until they successfully avoided virtual agents.

The experimental protocol included 3 blocks of trials. Each experimental block consisted of 8 conditions, i.e., four ST and four DT conditions, performed in a pseudorandom sequence, for a total of 24 trials. Cognitive ST were performed at the end of each block to adjust the retention time to the duration of the corresponding DT. Means of three trials were calculated for each variable and each experimental condition. The methodological choice to consider the averages of 3 trials instead of one trial was based on additional analyses (Supplementary Table S1). These showed that there was some variability between trials and that the average was probably more representative of true performance. While the general instructions given before the assessment included the description of all experimental conditions, no information about the condition (ST or DT) or the difficulty level of both tasks was shared with the participants before each trial. No prioritization instructions were given for the DT conditions.

To document potential side effects of VR, the Simulator Sickness Questionnaire [French version of the Cyberpsychology Lab of Université du Québec en Outaouais–UQO; based on Kennedy et al. (38)] was administered to the participants within minutes of completing the VR-based DT assessment. This questionnaire assesses 16 side effects using a 4-point scale (0 = none; 1 = slight; 2 = moderate; 3 = severe). The level of presence in virtual environment (VE) was measured using the Presence Questionnaire [French version of the Presence Questionnaire; Cyberpsychology Lab of UQO, 2002; based on Witmer and Singer (39)]. This questionnaire consists of 22 items, rated on a 7-point scale, about realism, possibility to act, quality of interface, possibility to examine, self-evaluation of performance, and sounds. The higher the score, the greater the sense of presence.

### Data Collection and Analyses

Locomotor performance was quantified with walking speed and minimal distance between the participant and the virtual agent during the avoidance for the complex locomotor task. These variables were calculated based on the participant and virtual agent position data in the VE, sampled at a frequency of 90 Hz. Participant and virtual agent position data were processed with a fourth-order Butterworth (6 Hz cut-off frequency) before calculating participant's walking speed, as the derivative with time, and the minimal distance between the participant and virtual agent positions during avoidance.

Cognitive accuracy was characterized by the number of items correctly recalled at the end of each trial for either the original (simple) or modified (complex) lists.

To quantify changes in locomotor and cognitive performance (in % of mean ST performance), DT costs (DTC) were calculated for each variable with the following formula:


$$\begin{array}{l} \text{DTC}=[(\text{mean ST performance}–\text{mean DT performance})/\\ \text{mean ST performance}]{\;}^{*}100. \end{array}$$

A positive walking speed DTC indicates that walking speed decreased in DT condition. A greater distance between the participants and virtual agents in ST condition compared to DT condition was indicated by a positive DTC. Furthermore, a positive cognitive DTC means better cognitive performance in ST condition compared to DT condition.

Data processing was performed using custom-made scripts written in MATLAB R2018b (The MathWorks, Inc., Natick, Massachusetts, USA) and Microsoft Excel 16.24 (Microsoft Corporation, Redmond, Washington, USA).

### Statistical Analyses

All statistical tests were performed using IBM SPSS Statistics 27.0 (SPSS Inc., Chicago, Illinois, USA). Non-parametric descriptive statistics were used to characterize participant and experimental variables (sociodemographic and clinical data, DTC). Before performing analyses to achieve the two objectives of the study, clinical profiles (locomotor, balance and cognitive functions) of the two experimental groups were compared with Mann-Whitney tests. To achieve the first objective, one sample Wilcoxon single-rank tests were performed to identify the presence of locomotor and cognitive DT interference in stroke survivors and subsequently determine a DTC pattern (mutual interference, cognitive interference, motor interference, no interference) for each combination of tasks. For the second objective, both DTC pattern and magnitude were compared between groups. After identifying DTC patterns in healthy adults (based on one-sample Wilcoxon single-rank tests, as in stroke survivors), we determined whether the observed DTC patterns were the same or different between the groups for each combination of tasks. Mann-Whitney tests were performed to compare all locomotor and cognitive DTC magnitude between the two groups. For all statistical tests, significance level was set at 0.05.

## Results

### Participant's Characteristics

Twelve community-dwelling chronic stroke survivors (5 women), as well as twelve age- and gender-matched healthy participants (5 women) were recruited. Participant's characteristics are described in Table 1. Although stroke survivors had good functional locomotor abilities, differences were observed in their locomotor, cognitive and balance abilities when compared to the healthy participants. Stroke participants walked more slowly and had more limited endurance than the control group. In addition, stroke participants had poorer balance and were less confident in their ability to perform various activities without losing their balance. However, no difference was observed between the two groups on the TUG-DTC (*p-value* = 0.410), which clinically measures DT locomotor abilities. Regarding cognitive measures, healthy individuals had better overall cognitive abilities as measured with the MoCA (*p-value* = 0.028). When looking specifically at short-term memory (measured with the MoCA memory subscale), cognitive inhibition and flexibility (D-KEFS subscales), no group differences were observed (*p-values* from 0.089 to 0.671), however. Not surprisingly, greater variability in stroke survivor scores was observed in both D-KEFS subscales compared with healthy participants.

**Table 1:** Participant's characteristics.

	**Participants with stroke (*n* = 12)**	**Healthy participants (*n* = 12)**	**Mann-Whitney test**
	**Median (25^th^-75^th^ percentiles)**	**Median (25^th^-75^th^ percentiles)**	***p*** **-value**
Age (years)	60.50 (53.50; 65.75)	61.00 (56.50; 63.75)	*p* = 0.843
Time since stroke (months)	13.41 (5.34; 48.90)	-	-
Type of stroke (ischemic / hemorrhagic)	10/2	-	-
Side of lesion (right / left/ bilateral)	7/2/3	-	-
**Clinical locomotor assessments**			
10MWT preferred speed (m/s)	1.10 (1.01; 1.16)	1.47 (1.38; 1.61)	***p*** **< 0.001**
10MWT maximal speed (m/s)	1.46 (1.29; 1.71)	1.99 (1.89; 2.19)	***p*** **< 0.001**
6MWT (m)	453.50 (408.25; 552.50)	638.50 (588.25; 675.75)	***p*** **< 0.001**
**Clinical DT assessment**			
TUG–DTC (%)	−25.44 (-41.27;−8.90)	−17.30 (-28.42;−7.07)	*p* = 0.410
**Balance assessments**			
Mini BESTest (max.: 28 pts)	23.50 (21.25; 25.00)	26.00 (25.00; 27.00)	***p*** **= 0.006**
ABC Scale (max.: 100%)	87.51 (71.64; 90.86)	96.88 (94.54; 99.36)	***p*** **< 0.001**
**Cognitive assessments**			
MoCA (max.: 30 pts)	26.00 (24.00; 28.75)	28.00 (27.00; 29.75)	***p*** **= 0.028**
Memory subscale (without any cues; max.: 5 pts)	3.5 (1.5; 5.0)	4.0 (4.0; 5.0)	*p* = 0.319
D-KEFS: Trail Making test (max.: 19 pts)	10.00 (6.00; 11.75)	11.00 (10.00; 13.00)	*p* = 0.089
D-KEFS: Color-Word Interference test (max.: 19 pts)	10.00 (6.75; 13.00)	11.50 (10.00; 12.00)	*p* = 0.671
**Reintegration to normal living**			
RNLI (max.: 22 pts)	1.00 (0.00; 3.75)	-	*-*
**VR-related questionnaires**			
Simulator Sickness Questionnaire (max.: 3 pts)	0.28 (0.15; 0.63)	0.22 (0.08; 0.38)	*p* = 0.347
Presence Questionnaire (max.: 7 pts)	5.37 (4.97; 5.79)	5.30 (4.61; 5.82)	*p* = 0.843

*Significant p-values (p < 0.05) are in bold. 6MWT, 6-minute Walk Test; 10MWT, 10-meter Walk Test; ABC Scale, Activities-Specific Balance Confidence Scale; D-KEFS, Delis-Kaplan Executive Functions System; Mini BESTest, Mini Balance Evaluation Systems Test; MoCA, Montreal Cognitive Assessment; RNLI, Reintegration of Normal Living Index; TUG-DTC, Timed-Up Go dual-task cost*.

As indicated by the median RNLI score of 1.0 (0.0; 3.75), stroke survivors in this sample considered themselves well reintegrated into their normal life.

Due to fatigue or cybersickness, not all participants completed the 3-block experimental protocol, but they all completed at least one block which allowed measurement of locomotor and cognitive DTCs in all DT conditions. Among stroke survivors, four participants completed all 24 trials in the virtual environment. The median number of trials completed was 14.5 trials (range: 8 to 24). Uncompleted trials were due to physical or cognitive fatigue. Among healthy participants, three did not complete all the trials (median: 24, range: 16 to 24). Two stopped because of cybersickness and one because of physical fatigue.

No difference between groups was observed regarding VR side effects (*p-value* = 0.347). Both groups appeared to tolerate VR well except for one healthy participant. In addition, no difference on level of presence (*p-value* = 0.843) was found between stroke survivors (5.37/7-4.97; 5.79) and healthy controls (5.30-4.61; 5.82).

### Locomotor and Cognitive DTC Magnitude and Patterns in Stroke Survivors

Despite a large variability within the stroke group, participants presented locomotor or cognitive DTCs in all DT conditions, except when combining the two simple tasks (walking forward without virtual agent avoidance and remembering a 5-item shopping list without modification; *p-values* from 0.332 to 0.937; Table 2). In the three other DT conditions, interferences in cognitive accuracy were measured (from 10.56 to 33.33 %; *p-values* from 0.002 to 0.037). In addition, DTCs in both locomotor outcomes were also noted in the most complex DT condition (6.54 and 10.78%; *p-values* 0.010 and 0.006). Thus, two patterns of DT were identified in stroke survivors. When DT conditions combined a simple and a complex task, cognitive interference was noted. When two complex tasks were performed simultaneously, mutual interference was observed in stroke survivors.

**Table 2:** Locomotor and cognitive DTCs in each DT condition for each group.

			**Cognitive tasks**				
				**5-item list**		**5-item list with modifications**	
				**DTC (%)**	***p*** **-value**	**DTC (%)**	***p*** **-value**
				**Median (25^th^-75^th^ percentiles)**		**Median (25^th^-75^th^ percentiles)**	
**Locomotor tasks**	**Forward walking**	Walking Speed	Stroke	−0.90 (-6.38; 7.40)	0.937	2.89 (-4.19; 12.25)	0.272
			Control	2.12 (-3.85; 3.54)	0.695	3.30 (-1.94; 10.10)	0.136
		Cognitive accuracy	Stroke	3.57 (-10.12; 28.63)	0.332	33.33 (11.46; 41.52)	**0.003**
			Control	3.34 (0.00; 12.50)	0.182	8.01 (-9.38; 39.62)	0.139
	**Walking with virtual agents**	Walking Speed	Stroke	−0.12 (-4.21; 5.32)	0.814	6.54 (2.03; 9.73)	**0.010**
			Control	−1.23 (-4.72; 4.87)	0.530	8.03 (4.71; 10.03)	**0.006**
		Minimal Distance	Stroke	3.71 (-9.73; 18.13)	0.433	10.78 (5.21; 20.78)	**0.006**
			Control	−1.19 (-2.52; 8.49)	0.638	5.03 (-3.63; 14.81)	0.099
		Cognitive accuracy	Stroke	10.56 (0.00; 34.48)	**0.037**	32.39 (22.92; 42.56)	**0.002**
			Control	6.67 (0.00 ; 10.00)	0.096	19.88 (9.94 ; 31.67)	**0.004**

*Significant p-values are in bold*.

### Comparisons in DT Abilities Between Stroke Survivors and Healthy Participants

When comparing the DT abilities of stroke survivors to those of age- and gender-matched healthy participants, differences in DTC patterns (Table 2) and magnitude (Table 3) were found. Firstly, whereas stroke survivors presented DT interference in all but the easiest DT condition, healthy participants exhibited DT interferences only when performing the DT condition combining the two complex tasks. Similar to stroke survivors, mutual interference was observed when healthy participants performed the most complex DT condition. However, stroke survivors presented cognitive interferences in two DT conditions, while this DTC pattern was not observed in healthy participants. Secondly, when comparing DTC magnitude between groups, only one significant difference was highlighted. The stroke group had a greater DTC in cognitive accuracy than the control group [U = 112.00; *p-value* = 0.020; effect size (r) = 0.47] in the most complex DT condition.

**Table 3:** DTC comparisons between groups.

			**Cognitive tasks**			
			**5-item list**		**5-item list with modifications**	
			**Mann Whitney**		**Mann Whitney**	
			**U**	***p*** **-value**	**U**	***p*** **-value**
**Locomotor tasks**	**Forward walking**	Walking speed	72.00	1.00	71.00	0.977
		Cognitive accuracy	75.00	0.887	95.50	0.160
	**Walking with virtual agents**	Walking Speed	79.50	0.671	54.00	0.319
		Minimal distance	77.00	0.799	98.00	0.143
		Cognitive accuracy	95.50	0.178	112.00	**0.020**

*Significant p-values are in bold*.

## Discussion

In this pilot study, locomotor and cognitive DT abilities in community-dwelling stroke survivors were quantified as they walked along a corridor in a virtual shopping mall and simultaneously memorized a 5-item shopping list. These abilities were then compared to those measured in age- and gender-matched healthy participants. Stroke survivors presented DT interferences in all DT conditions except the simplest one. Cognitive interference was noted when they have performed DT conditions combining a simple and a complex task. Furthermore, mutual interference (both locomotor and cognitive interference) was observed when they have been exposed to the DT condition including both complex tasks. Different results in DTC patterns were obtained in healthy individuals. Indeed, age-matched healthy individuals showed no DT interference except for the most complex DT conditions. Similar to stroke participants, healthy individuals exhibited a mutual interference when performing the most complex DT condition. When comparing the magnitude of locomotor and cognitive DTCs between groups, a difference was measured in cognitive accuracy when they had to avoid virtual agents and remember the shopping list with modifications. Indeed, stroke survivors exhibiting a greater DTC in cognitive accuracy. No other differences between groups were found in DTC magnitude.

As already observed in previous studies, several patterns of interference (9) can be observed when stroke survivors are asked to perform two activities simultaneously [reviewed in (18)]. In the present study, stroke survivors presented cognitive interference or mutual interferences depending on the complexity of locomotor and cognitive tasks. Interference in cognitive performance without any deterioration in locomotor performance may suggest that stroke survivors prioritized the locomotor task (40). This “posture-first strategy” has been previously observed in stroke survivors with DT involving a simple walking task (41), as well as in DT involving obstacle crossing (42, 43). This strategy is known to be adopted when the presence of sensory or motor impairments alter responses or adaptations to physical perturbations during walking, or when individuals feel that performing locomotor tasks could put them at risk (44). In the present study, it is likely that participants with stroke had to pay more attention to their posture and balance while walking due to their lesion-induced locomotor and balance disorders. In addition, a certain level of uncertainty was present for each trial since no prioritization instructions were given to the participants nor any information about the upcoming conditions. The presence of moving obstacle to avoid could also have potentially heightened participant's perception of a riskier situation. Conscious or not, this prioritization of locomotor tasks could be considered as a coping strategy to improve safety (45). On the other hand, the absence of significant DTC in locomotor outcomes could also be explained by a large within-group variability in the results of stroke survivors. Different strategies seem to have been used during DT assessment. For instance, some participants decreased, as expected, their walking speed in DT conditions compared to ST conditions (i.e., positive DTC) whereas others opted for the opposite strategy (negative DTC). Although initially unexpected as a result, this increase in walking speed could be explained by the participant's desire to reduce the retention time of the memory task. An increased walking speed was similarly observed when stroke survivors were asked to perform a similar task involving the memorization of shopping list items while walking on a self-paced treadmill (21).

When stroke survivors were required to perform the most complex DT condition, cognitive and locomotor DTCs were noted, suggesting that stroke survivors were not able to maintain their locomotor performance as measured in ST. This pattern of interference has been previously documented in stroke survivors, especially when performing a DT including the negotiation of obstacles while walking (18). Considering that complex conditions seem to be the most representative of community walking (46), this result suggests that individuals who sustained a stroke may exhibit mutual interference in their everyday life. This experimental condition was the only one to induce DT interference in age-matched healthy individuals. Similar to stroke survivors, they presented mutual interference. However, the magnitude of this interference was greater in those who had a stroke. Presence of interferences when performing two tasks simultaneously demonstrates that both performances require common cognitive resources (7, 47). Thus, the results on DT patterns highlight the fact that conflicts in limited cognitive processes when dual tasking were more frequent in stroke survivors than in healthy participants. Due to the presence of multiple impairments, stroke survivors appear to make greater demands on their cognitive resources during locomotor tasks, thereby increasing the incidence of interference during cognitive-locomotor DT conditions (48). From a mechanistic perspective, a few studies have demonstrated that performing an additional task while walking increases prefrontal cortex (PFC) activity (49–52). This increase in PFC activation is greater in stroke survivors than in healthy young adults (51), while results from comparisons with older adults were equivocal (49, 51).

It is noteworthy that the results of the present pilot study were obtained in stroke survivors who reported being able to walk in the community when they felt it necessary (as demonstrated by the RNLI) and who had a walking speed and endurance level above the prerequisites for an independent and safe community walk [walking speed of 0.8 m/s or higher and walking endurance of 300 m or higher (6, 53)]. Despite their good locomotor abilities, DT interferences were nonetheless observed in these participants. This finding underscores the importance of assessing locomotor AND cognitive DT abilities in all stroke survivors. It is reasonable to assume that individuals with more severe limitations following a stroke may be even more affected when performing DT activities in the community. Indeed, a recent study showed that stroke survivors with greater motor and balance impairments had greater locomotor DTCs than stroke survivors with fewer impairments when performing a cognitive-locomotor DT (54).

Whereas, between-group differences in DT abilities were highlighted using the VR-based DT assessment protocol, no difference was detected with a frequently-used DT clinical test, namely the TUG-DT. This discrepancy appears to be explained by the fact that TUG-DTC only quantifies the impact of DT on walking performance without considering cognitive performance. The TUG-DT may therefore not be sufficient to assess DT abilities in a comprehensive manner. Indeed, cognitive DT abilities should always be investigated when evaluating DT abilities. Cognitive DTC measured with the VR-based ecological protocol appears to be an interesting measure to distinguish the DT abilities of stroke survivors from those of healthy individuals. As such, a VR-based protocol appears to be an interesting option for assessing DT abilities in stroke survivors, although accessibility to such technology may still be limited in clinical practice.

## Study Limitations

Some limitations to the pilot study must be noted. While the omnidirectional platform allows to assess DT abilities during activities representative of daily living, there are differences in locomotor patterns between overground walking and walking on this platform (55). Participants had to learn how to walk on the omnidirectional platform and were thus provided two familiarization periods: one at the end of their first visit and another one at the beginning of the second visit. This familiarization helped to limit learning effects. Furthermore, all conditions (ST and DT) within the two groups were performed with the same equipment. Nonetheless, the results should be interpreted with caution considering that a newly learned walking pattern may increase the demands on attentional processes compared to normal overground walking. To facilitate lower limb movements on the platform, participants had the option of leaning slightly on the ring. However, the weight put on the ring was not quantified and could therefore vary across participants. Another limitation is related to the fact that not all stroke survivors were able to complete the entire VR-based DT assessment protocol (24 trials) due to physical or cognitive fatigue. To mitigate these effects, participants were asked about their level of fatigue after each trial and allowed to take breaks. The assessment was stopped when fatigue persisted despite breaks. Additional analyses, however, showed that the comparative results between the groups were the same when DTCs were calculated from the first trial only or from the average of all completed trials (Supplementary Table S2). It is therefore reasonable to believe that differences in the number of trials between the groups did not affect the results of comparisons. Furthermore, completing the trials in a pseudo-randomized order also helped to limit learning and fatigue effects. The order of the trials is considered “pseudo-random” since it was necessary to impose the cognitive ST trials at the end of each block (the last 2 trials out of a total of 8) to ensure the same retention time between DT and ST conditions. It is possible that the performance in these last trials (cognitive ST) was improved due to a learning effect and, consequently, the DTC magnitude would be accentuated. It should be noted, however, that a different list of items was proposed on each trial and each list related to a certain type of store (e.g., grocery, pharmacy, hardware store, clothing store), thus limiting the potential learning effects.

## Conclusions

Stroke survivors exhibited DT interferences while performing DT activities representative of daily living in an ecological virtual environment. This population seemed to prioritize locomotor task (cognitive interference) until they are asked to execute the most complex task. In this condition, it was rather interferences in both locomotor and cognitive performances that have been observed. Whereas, a frequently used clinical DT test failed to distinguish the DT abilities of stroke survivors from those of age-matched participants, the VR-based DT assessment protocol highlighted group differences in the cognitive DTC. Since DT can have an impact on locomotor and cognitive performances, it is crucial to assess both, during DT activities representative of daily living, to obtain a comprehensive picture of DT abilities in stroke survivors or elderly. Although this pilot study demonstrates the potential of such a virtual assessment protocol to quantify DT abilities in stroke survivors, future studies should be completed on its psychometric properties, as well as on its possible implementation in clinical settings.

## Data Availability Statement

The raw data supporting the conclusions of this article will be made available by the authors, without undue reservation.

## Ethics Statement

The studies involving human participants were reviewed and approved by Research Ethics Committee in Rehabilitation and Social Integration, CIUSSS de la Capitale-Nationale, Quebec City, Canada. The patients/participants provided their written informed consent to participate in this study.

## Author Contributions

AD-B, AL, BM, M-CO, and AB designed the experiment. AD-B participated in the participant's recruitment and completed all data collection. AD-B, AL, BM, and AB contributed to the analysis and interpretation of the data. AD-B and AB wrote the first draft of the manuscript. All authors read and approved the final manuscript.

## Funding

This work was supported by the Team of researchers in Immersive Technology in Rehabilitation (ITR) of the Centre for Interdisciplinary Research in Rehabilitation and Social Integration (Cirris). AD-B received PhD grants from the Cirris, the Fonds de recherche et d'enseignement de la Faculté de médecine de l'Université Laval, and the Fonds de Recherche du Québec-Santé (FRQ-S). The funding bodies had no role in the design of the study, data collection and analysis, interpretation of data, and in writing the manuscript.

## Conflict of Interest

The authors declare that the research was conducted in the absence of any commercial or financial relationships that could be construed as a potential conflict of interest.

## Publisher's Note

All claims expressed in this article are solely those of the authors and do not necessarily represent those of their affiliated organizations, or those of the publisher, the editors and the reviewers. Any product that may be evaluated in this article, or claim that may be made by its manufacturer, is not guaranteed or endorsed by the publisher.
