# Supplementary material for: Dual-Task Abilities During Activities Representative of Daily Life in Community-Dwelling Stroke Survivors: A Pilot Study
Source: Front Neurol. 2022 May 3;13:855226. doi: 10.3389/fneur.2022.855226 (PMC9110886; doi:10.3389/fneur.2022.855226)
Supplement: Supplementary file 1 [file Table_1.pdf]

Supplementary Material

Supplementary Table 1: Comparisons between the DTCs calculated from the first trial only and the average of all completed trials.

LOCOMOTOR TASKS					COGNITIVE TASKS			
					**5-item list**		**5-item list with modifications**	
					DTC (%)	*p-value*	DTC (%)	*p-value*
					Median (25th-75th percentiles)		Median (25th-75th percentiles)	
**Forward walking**	Walking Speed	Stroke	*Mean of all trials*		-0.90% (-6.38; 7.40)	.937	2.89% (-4.19; 12.25)	.272
			First trial		0.36% (-4.04; 7.09)	.754	2.82% (-1.03; 15.57)	.084
		Control	*Mean of all trials*		2.12% (-3.85; 3.54)	.695	3.30% (-1.94; 10.10)	.136
			First trial		-9.76% (-16.18; -0.20)	.071	6.01% (.87; 17.9)	.071
	Cognitive accuracy	Stroke	*Mean of all trials*		3.57 (-10.12; 28.63)	.332	33.33 (11.46; 41.52)	**.003**
			First trial		0% (0.0; 35.0)	.233	25% (0.0; 33.33)	**.023**
		Control	*Mean of all trials*		3.34 (0.00; 12.50)	.182	8.01 (-9.38 ; 39.62)	.139
			First trial		.00% (.00; 20.00)	**.046**	.00% (.00; 23.75)	.351
**Walking with virtual agents**	Walking Speed	Stroke	*Mean of all trials*		- 0.12 (-4.21; 5.32)	.814	6.54 (2.03 ; 9.73)	**.010**
			First trial		-.99% (-6.69; 7.11)	.937	10.44% (3.70; 16.90)	**.012**
		Control	*Mean of all trials*		-1.23 (-4.72 ; 4.87)	.530	8.03 (4.71 ; 10.03)	**.006**
			First trial		0.49 (-8.17; 8.24)	.937	17.50 (-1.92; 23.94)	.117
	Minimal Distance	Stroke	*Mean of all trials*		3.71 (-9.73 ; 18.13)	.433	10.78 (5.21 ; 20.78)	**.006**
			First trial		6.59% (-14.86; 19.90)	.695	15.17% (-.30; 29,24)	**.041**
		Control	*Mean of all trials*		-1.19 (-2.52 ; 8.49)	.638	5.03 (-3.63 ; 14.81)	.099
			First trial		10.45 (-.90; 15.03)	.099	9.33 (-4.17; 22.98)	.209
	Cognitive accuracy	Stroke	*Mean of all trials*		10.56 (0.00 ; 34.48)	**.037**	32.39 (22.92 ; 42.56)	**.002**
			First trial		26.67% (0.0; 47.50)	**.038**	50% (27.08; 66.67)	**.002**
		Control	*Mean of all trials*		6.67 (0.00 ; 10.00)	.096	19.88 (9.94 ; 31.67)	**.004**
			First trial		10.00 (.00; 20.00)	**.024**	22.50 (5.00; 40.00)	**.007**

Significant *p-values* are in bold.

Supplementary Table 2 : DTC comparisons between groups when calculated on the first trial only and on the average of all completed trials.

				**COGNITIVE TASKS**			
				**5-item list**		**5-item list with modifications**	
				Mann Whitney		Mann Whitney	
				U	p-value	U	p-value
**LOCOMOTOR TASKS**	**Forward walking**	Walking Speed	*Mean of all trials*	72.00	1.00	71.00	.977
			First trial	101.00	.101	67.00	.799
		Cognitive accuracy	*Mean of all trials*	75.00	.887	95.50	.160
			First trial	76.00	.843	94.50	.198
	**Walking with virtual agents**	Walking Speed	*Mean of all trials*	79.50	.671	54.00	.319
			First trial	68.00	.843	58.00	.443
		Minimal Distance	*Mean of all trials*	77.00	.799	98.00	.143
			First trial	69.00	.887	87.00	.410
		Cognitive accuracy	*Mean of all trials*	95.50	.178	112.00	**.020**
			First trial	84.00	.514	122.00	**.003**

Significant *p-values* are in bold.
